# Awake Major Abdominal Surgeries in the COVID-19 Era

**DOI:** 10.1155/2021/8763429

**Published:** 2021-02-22

**Authors:** Andrea Romanzi, Nicola Boleso, Giuseppe Di Palma, Davide La Regina, Francesco Mongelli, Maria Milanesi, Antonella Putortì, Fabrizio Rossi, Roberta Scolaro, Michel Zanardo, Alberto Vannelli

**Affiliations:** ^1^Department of General Surgery, Valduce Hospital, Como 22100, Italy; ^2^Department of Anesthesiology and Critical Care, Valduce Hospital, Como 22100, Italy; ^3^Department of General Surgery, Regional Hospital of Bellinzona and Valli, Bellinzona 6500, Switzerland

## Abstract

**Background:**

During the outbreak of coronavirus disease 2019 (COVID-19), allocating intensive care beds to patients needing acute care surgery became a very difficult task. Moreover, since general anesthesia is an aerosol-generating procedure, its use became controversial. This strongly restricted therapeutic strategies. Here, we report a series of undeferrable surgical cases treated with awake surgery under neuraxial anesthesia. Contextual benefits of this approach are deepened.

**Methods:**

During the first pandemic surge, thirteen patients (5 men and 8 women) with a mean age of 80 years, needing undelayable surgery due to abdominal emergencies, underwent awake open surgery at our Hospital. Prior to surgery, all patients underwent nasopharyngeal swab tests for COVID-19 diagnosis. In all cases, regional anesthesia (spinal, epidural, or combined spinal-epidural anesthesia) was performed. Intraoperative and postoperative pain intensities have been monitored and regularly assessed. A distinct pathway has been set up to keep patients of uncertain COVID-19 diagnosis separated from all other patients. Postoperative course has been examined.

**Results:**

The mean operative time was 87 minutes (minimum 60 minutes; maximum 165 minutes). In one case, conversion to general anesthesia was necessary. Postoperative pain was always well controlled. None of them required postoperative intensive care support. No perioperative major complications (Clavien–Dindo ≥3) occurred. Early readmission after surgery never occurred. All nasopharyngeal swabs resulted negative.

**Conclusions:**

In our experience, awake laparotomy under regional anesthesia resulted feasible, safe, painless, and, in specific cases, was the only viable option. This approach allowed prevention of the need of postoperative intensive monitoring during the COVID-19 era. In such a peculiar time, we believe it could become part of an ICU-preserving strategy and could limit viral transmission inside theatres.

## 1. Introduction

During the outbreak of coronavirus disease 2019 (COVID-19), the demand for critical care beds among the medical services has rapidly exceeded their supply. The absolute anomaly of this pandemic put almost all health care systems to the test. Consequently, elective surgery has been drastically limited. Moreover, with such a background, allocating intensive care beds to patients needing acute care surgery was even a more difficult task as they fulfilled the criteria for postoperative intensive care.

Major abdominal procedures are generally carried out with minimally invasive surgery (MIS) under general anesthesia (GA). The Surgical Community has focused its attention on the potential MIS-related risks of contagion so that its use in the operating theatre has become controversial. Furthermore, old frail patients are characterized by decreased reserve and diminished resistance to stressors. On these patients, exposure to the stress of surgery under GA is associated with increased risk of adverse outcomes and higher levels of resource use [1].

In 2019, eight frail patients needing emergency abdominal surgery underwent awake laparotomy under regional anesthesia (ALURA) at our Hospital. Considering their frail cardiovascular and respiratory reserves, pneumoperitoneum and GA would have presumably increased morbidity and mortality. Given this, in all cases, regional anesthesia (RA) was performed. On our preliminary experience, ALURA resulted feasible, safe, and painless [2, 3]. In selected patients, this approach represented a valid alternative to GA.

Bearing this in mind, at the beginning of the COVID-19 era, we wondered if ALURA would have resulted functional to perform undeferrable surgeries in selected cases, limiting the contagion risk in the operating room and, additionally, the number of postoperative ICU admissions. Our early experience and results are reported.

## 2. Methods

From February 11^th^ to May 20^th^ 2020, thirteen patients (5 men and 8 women) with a mean age of 80 years, needing undelayable surgery due to abdominal emergencies, underwent ALURA at the Department of Surgery of our Hospital (Table 1). All patients, preoperatively assessed by the anesthesiologist, were considered at high risk (ASA score ≥ 3). Twelve patients out of thirteen (except patient #9) were identified as “frail” because of their age (≥80 yo), multiple major comorbidities, or clinical conditions. These patients were considered unlikely to tolerate GA which, in the best case, would have seriously prolonged their stay in the ICU. Since we believe this approach may help to limit the contagion risk, patient #9 also underwent ALURA. The procedure was preoperatively explained to each patient.

All patients always wore an FFP2 mask inside the hospital, during all the phases of each surgical procedure and after the operation. Each patient filled in a preadmission screening questionnaire to assess the risk of a recent contagion. Each patient underwent the nasopharyngeal swab test for COVID-19 diagnosis preoperatively but was considered positive by healthcare professionals until proven otherwise, following our internal “pandemic-specific” flowchart. Patients with positive result at the preadmission screening questionnaire or having the result of their swab still pending underwent LA.

Preoperative blood tests included complete blood count (for platelets count) and basic coagulation tests (prothrombin time, INR, and activated partial thromboplastin time). In order to prevent bleeding complications, anticoagulants were suspended (at different times on the basis of the specific anticoagulant) when possible. If needed, bridging heparin therapy was introduced. The last administration of low-molecular-weight heparin (LMWH) was 8 hours prior to the procedure.

The healthcare personnel involved wore personal protective equipment (eye protection, FFP2 mask, long sleeved fluid repellent gown, gloves, and shoe covers) during all the phases of each surgical procedure.

Surgery was performed under combined spinal-epidural (CSE) anesthesia in 10 cases (needle sizes: 16G and 25G), under spinal anesthesia (SA) in 2 cases (needle size: 25G), and under epidural anesthesia (EA) in 1 case (needle size: 17G). At our Institution, CSE anesthesia is the standard RA technique. SA was preferred in 2 cases due to severe spine deformities. EA was performed in 1 case (the last of our case series) after we ran out of CSE sets since during the pandemic, all medical supplies showed considerable delays.

RA was performed by four different anesthesiologists, all having considerable expertise in SA, EA, and CSE anesthesia. Lumbar puncture was routinely performed by sitting the patient on the edge of the bed, with the legs resting on a stool. The patient was then asked to roll his/her shoulders and upper back forwards, and the stool was positioned to bring the thighs up towards the abdomen. Once the correct entry point was identified, the skin was cleaned with antiseptic and local anesthesia was performed (around 3 ml of lidocaine 20 mg/ml solution for injection). Although several CSE techniques are used in clinical practice (including the two-needle, two-interspace technique), the currently preferred technique for CSE anesthesia at our Institution is the needle-through-needle (NTN) technique. This includes use of separate epidural and spinal needles (B. Braun Eposcan^®^ kit for CSE anesthesia; B. Braun, Melsungen AG, Germany). The epidural space is located with a conventional epidural needle and technique, and then, a long spinal needle is passed through the epidural needle until cerebrospinal fluid appears in the hub of the spinal needle. Drug is administered via the spinal needle into the subarachnoid space, the spinal needle is removed, and finally an epidural catheter is inserted into the epidural space.

During EA, a bolus of Naropin 7.5 mg/ml and morphine sulfate 10 mg/ml solution has been injected in the epidural space around 15 minutes before surgical incision. During SA and CSE anesthesia, a bolus of hyperbaric bupivacaine 5 mg/ml and morphine sulfate 10 mg/ml solution has been injected in the subarachnoid space around 7 minutes before surgical incision. Composition of subarachnoid and epidural boli was calibrated on the basis of patients' age, height, and constitution.

All CSE techniques were performed using a catheter through a needle set which allowed placement of the epidural delivery system (EDS). After surgery, a solution of sterile water, Naropin 7.5 mg/ml, and morphine sulfate 10 mg/ml was injected through this system for postoperative analgesia. Naropin and morphine sulfate doses were calibrated on the basis of the patient constitution. The infusion speed was set on 2 or 4 ml/hour, on the same basis. After SA, an elastomeric pump could be placed for postoperative analgesia if needed. EDS and elastomeric pump were all removed on postoperative day (POD) 3 by appropriately trained nurses.

Procedures were performed by two different surgeons with broad experience in open surgery. Anesthesia was always assessed and judged adequate by pinprick before surgery started. In order to avoid aerosolization, cautery utilization was strongly limited, energy devices were used at their absolute lowest settings, and Kelly clamps and heavy ties were preferred to control mesentery when possible. Intraoperative and postoperative pain intensities have been monitored and regularly assessed through the use of the numeric rating scale (NRS).

Caprini score was used for individualized venous thromboembolism (VTE) prophylaxis after surgery [4]. This scoring system is able to risk-stratify patients for postoperative VTE [5]. LMWH was reintroduced at least 12 hours after surgery (if allowed by the patient comorbidities or clinical conditions) and taking surgical postoperative bleeding risk into account.

A distinct area was set up for postoperative recovery to keep patients having pending or uncertain COVID-19 diagnosis separated from all other patients. Patients were supposed to be moved to “clean” or “COVID-19-dedicated” wards on the basis of the tests' response. Patients were admitted to single rooms in case of doubt of recent anamnesis, or to double rooms if their recent anamnesis arose no index of suspect.

In absence of complications, blood test controls were scheduled on PODs 1, 3, and 7. At the beginning of May 2020, we retrospectively analyzed these data. We considered patients' medical history and operative results: surgical time, conversion to GA, LA-related complications, intraoperative blood transfusion, ICU admission, urinary catheter removal, first bowel movement (gas and faeces) after operation, early postoperative complications, postoperative length of stay (LOS), and readmission due to postoperative complications that occurred after discharge. The Clavien–Dindo classification was used to assess postoperative complications [6]. In case of multiple complications occurring in a single patient, the complication of higher grade was considered.

## 3. Results

LA-related complications never occurred. During all SA and CSE anesthesia, the solution injected into the subarachnoid space had the following composition: hyperbaric bupivacaine 5 mg/ml (minimum dose: 2 ml; maximum dose: 2.3 ml) and morphine sulfate 10 mg/ml (minimum dose: 100 mcg; maximum dose: 150 mcg). During EA, the solution injected into the epidural space had the following composition: Naropin 7.5 mg/ml (16 ml) and morphine sulfate 10 mg/ml (100 mcg).

Seven patients required sedation during surgery for a better discomfort control. Sedation was obtained through intravenous administration of midazolam 15 mg/3 ml (minimum dose: 1 mg; maximum dose: 2 mg) and propofol 10 mg/ml (minimum dose: 40 mg; maximum dose: 60 mg). The mean operative time was 87 minutes (minimum 60 minutes; maximum 165 minutes). In one case (7.7%), conversion to GA was necessary. This case was the surgery with the longest operative time (165 minutes). After EA and CSE anesthesia, a solution of sterile water (192 ml), Naropin 7.5 mg/ml (minimum dose: 100 mg; maximum dose: 150 mg), and morphine sulfate 10 mg/ml (minimum dose: 2 mg; maximum dose: 5 mg) was injected through EDS for postoperative analgesia. In one of the two SA, an elastomeric pump was placed for postoperative analgesia. This was filled with a solution of sterile water (60 ml), morphine sulfate (10 mg), and ondansetron (8 mg) at an infusion speed of 2 ml/hour. Postoperative pain, regularly assessed through NRS, always was well controlled. Only one patient (after EA) required postoperative intravenous administration of paracetamol 10 mg/ml (1 g every 8 hours, for 24 hours) because of NRS value higher than 3.

None of the patients required postoperative intensive care support. One patient required both preoperative and postoperative blood transfusion due to the severe anaemia (Hb < 7 mg/dl) caused by tumour bleeding. All preoperative nasopharyngeal swabs for COVID-19 diagnosis resulted negative.

One patient developed postoperative infection of the urinary tract requiring intravenous administration of targeted antibiotic therapy. This did not substantially prolong his postoperative LOS. Perioperative results are summarized in Table 2.

Patients were always discharged in the absence of postoperative symptoms (e.g., dyspeptic symptoms, abdominal pain, urinary disorders, fever, and laboratory abnormalities) and after first passage of stool. Mean time for urinary catheter removal was POD 3, mean time for the passage of first flatus was POD 3, mean time for first defecation was POD 5, and mean postoperative LOS was 6 days. We did not register any cases of early readmission after surgery (within 72 hours of discharge). Patient #2 was introduced to our observation with an indwelling catheter because he was affected with multiple sclerosis. He was not considered in the calculation of the mean time for urinary catheter removal.

## 4. Discussion

Generally, major abdominal operations are carried out with MIS (laparoscopy, robotic surgery). Besides this, technical advances and new drugs led to a progressive standardization of GA for major surgeries. MIS and GA are both aerosol-generating procedures, so when COVID-19 outbreak began, they became under great debate as they may contribute to spreading contamination inside operating theatres. Initial reports advised against their use [7–10]. Furthermore, while RA preserves patients' cardiorespiratory function, GA can be associated with delayed recovery after anesthesia and can lead to the admission of the patient to the ICU.

Keeping all this into account, despite the well-known benefits of MIS and GA, we had to consider the higher contagion risk and to balance pros and cons. For these reasons, British colleagues recently warned the Surgical Community, supporting and encouraging the increased use of regional anesthesia during the pandemic [11].

During the pandemic, hospitals halted or drastically limited surgery, not only to limit spreading contagions but also to preserve ICU beds, personnel, and equipment for critically ill COVID-19 patients [2]. At the moment, the presence of COVID-19 patients into the ICU is decreased. Considering all this, allocating intensive care beds to emergency cases or to elective high-risk cases is still complex and sometimes unfeasible. All hospitals then organized dedicated protocols and workforce training [3].

In our experience, several undeferrable surgical cases were treated through the association of neuraxial anesthesia and laparotomic surgery (when nonoperative management was inapplicable) without resorting to ICU admission. Conversion to GA occurred in one single case. Thanks to our previous experience on this “awake approach,” we were able to consider and to perform the surgical treatment even when no ICU beds were available. This avoided the frustrating, and often unsuccessful, research by telephone of ICU availability at other nearby hospitals. Moreover and most importantly, we were able to avoid delicate transfers. Especially at the beginning of the pandemic, arranging transports for patients of uncertain COVID-19 diagnosis was troublesome for many institutions, for several reasons. Multiple advantages on the whole management of acute care surgery-needing patients were then observed to be related to ALURA.

In the operating room, RA limited the use of the ventilator and the following replacement of the high-efficiency particulate air (HEPA) filter. Apart from the limited use of cautery and energy devices, ALURA did not require any significant modifications of the surgical technique. Nevertheless, although RA did not entail a relevant elongation of the operative time, it caused discomfort to some patients who became intolerant to the long procedure. To this purpose, seven patients required sedation. Our early experience did not highlight a correlation between the height of the block and the incidence of sedation. In fact, the higher block of our case series (SA at L1-L2 for right colectomy; patient #9) required sedation. On the contrary, the higher colon mobilization (EA at L2-L3 for transverse colon resection; patient #13) did not.

Postoperatively, patients' pain was always well controlled. The limited administration of opioids maximized RA benefits: adequate pain control, short-lasting paralytic ileus, and mild nausea and vomiting. Epidural analgesia-associated early recovery of intestinal peristalsis has been supposed to be a risk factor for anastomotic leakage [12]. In our experience, this never occurred.

Compared to our average LOS, this approach did not show to be related to prolonged postoperative courses nor to higher rates of readmissions after surgery. The longest postoperative LOSs were observed in older and more frail patients, apparently independently of the severity of the clinical picture that required surgical treatment. Further studies are needed to clarify this aspect.

Although MIS under GA is frequently advocated for acute care surgery, we strongly deem that open surgery under RA should be taken into consideration whenever surgery is planned for any patient who poses an infection risk. In such an unstable and evolving organizational setup, ALURA may also result crucial in other delicate cases such as postneoadjuvant abdominal surgery or borderline resectable cancers, when postponing cancer care is unacceptable. Moreover, RA appears as a precious alternative to GA for patients presenting frail cardiovascular and respiratory reserves and in whom GA would presumably increase morbidity and mortality [13].

Finally, as the major Societies of Surgeons and Anesthesiologists suggested, preserving healthcare resources and manpower is paramount; therefore, developing a distinct perioperative pathway for the patients of uncertain COVID-19 diagnosis is essential for every hospital [14].

This study is based on a retrospective single-centre experience, carried out on a small single group of patients due to the peculiar social and historical background. At the moment, this precluded any statistical analyses. Considering all these limitations, our results are supportive but not conclusive. Further data derived from randomized controlled trials including a larger number of patients are needed to examine this approach in depth (e.g., to compare potential advantages, in terms of ICU admissions and anesthesiological complications to laparoscopy under general anesthesia). Unfortunately, only a few case reports and even fewer case series deepening the influence of RA on the outcomes after acute care surgery are available [15–18]. As known, RA techniques slightly differ from institution to institution [19, 20]. This impedes the standardization of the technique and limits the comparison between different studies. Coordinated multicentre studies would help in identifying a common protocol and allow for a deeper investigation of the applications, advantages, and limitations of this approach.

Our present experience suggests that, in support to open surgery, RA may help to limit the intubation-related risk of contagions inside theatres. In the COVID-19 era, neuraxial anesthesia for awake surgery should always be considered as a functional option, for selected patients. Especially in prevision of future congestions of the ICU network, we believe this approach could become part of an ICU-preserving strategy allowing surgeons to carry out undeferrable surgeries.

## Figures and Tables

**Table 1:** Patients' clinicopathological characteristics.

Patient	Age	Sex	Diagnosis	Comorbidities						
				C	P	DM	L	R	O	PAS
1	91	M	Obstructing rectal cancer	x	x					x
2	74	M	Covered cacum cancer perforation with local abscess	x	x	x	x		x	
3	88	M	Bleeding right colon cancer	x	x	x	x		x	x
4	88	F	Obstructing right colon cancer	x		x		x		x
5	89	M	Acute perforated appendicitis	x						x
6	93	F	Bleeding rectal cancer	—						x
7	78	F	Bleeding right colon cancer	x	x		x			x
8	78	F	Obstructing sigmoid colon cancer	x						x
9	69	F	Bleeding right colon cancer	—						x
10	82	F	Intestinal obstruction in strangulated right crural hernia	x					x	
11	81	M	Bleeding right colon cancer	x		x	x	x		x
12	70	F	Intestinal obstruction in advanced ovarian cancer	x			x		x	x
13	68	F	Iatrogenic transverse colon ischaemia after bilateral ovariectomy for bilateral ovarian cancer						x	X

C: cardiovascular disease; DM: diabetes mellitus; L: liver disease; O: other minor comorbidities; P: pulmonary disease; PAS: previous abdominal surgery; R: end-stage renal disease.

**Table 2:** Regional anesthesia and perioperative results.

Patient	Anesthesia	Lumbar puncture	Sedation	Surgery performed	Operative time (min)	Conversion to GA	PO complications	PO blood transfusion
1	CSE	L2-L3	Yes	LAR	120	—	—	—
2	CSE	L2-L3	Yes	RC	120	—	—	—
3	CSE	L2-L3	Yes	RC	65	—	—	—
4	CSE	L2-L3	—	RC	60	—	—	—
5	CSE	L2-L3	—	CR	60	—	—	—
6	CSE	L2-L3	—	LAR	150	—	—	—
7	CSE	L2-L3	—	RC	60	—	—	Yes
8	CSE	L2-L3	Yes	SR	165	Yes	—	—
9	SA	L1-L2	Yes	RC	60	—	—	—
10	SA	L2-L3	—	IR + HP	60	—	CD2	—
11	CSE	L3-L4	Yes	RC	70	—	—	—
12	CSE	L3-L4	Yes	ExLAP + CS	70	—	—	—
13	EA	L2-L3	—	TR	75	—	—	—

CD: Clavien–Dindo; CR: cecum resection; CS: cytoreductive surgery; CSE: combined spinal-epidural anesthesia; EA: epidural anesthesia; ExLAP: exploratory laparotomy; GA: general anesthesia; HP: hernioplasty; IR: ileal resection; LAR: low anterior resection; min: minutes; PO: postoperative; RC: right colectomy; SA: spinal anesthesia; SR: sigmoid resection; TR: transverse colon resection.

## Data Availability

Data are available and may be requested at Valduce Hospital, Via Dante Alighieri, 11 22100 (Como), Italy +39 031 324111 (direzione.sanitaria@valduce.it).
